# Anti-*Campylobacter* Probiotics: Latest Mechanistic Insights

**DOI:** 10.1089/fpd.2022.0039

**Published:** 2022-10-12

**Authors:** Igori Balta, Eugenia Butucel, Lavinia Stef, Ioan Pet, Gratiela Gradisteanu-Pircalabioru, Carmen Chifiriuc, Ozan Gundogdu, David McCleery, Nicolae Corcionivoschi

**Affiliations:** ^1^ Bacteriology Branch, Veterinary Sciences Division, Agri-Food and Biosciences Institute, Belfast, United Kingdom.; ^2^ Faculty of Animal Science and Biotechnologies, University of Agricultural Sciences and Veterinary Medicine, Cluj-Napoca, Romania.; ^3^ Faculty of Bioengineering of Animal Resources, Banat University of Agricultural Sciences and Veterinary Medicine—King Michael I of Romania, Timisoara, Romania.; ^4^ Research Institute of University of Bucharest, Bucharest, Romania.; ^5^ Department of Infection Biology, Faculty of Infectious and Tropical Diseases, London School of Hygiene and Tropical Medicine, London, United Kingdom.

**Keywords:** probiotics, *Campylobacter* spp, mechanisms, poultry, humans

## Abstract

The *Campylobacter* genus is the leading cause of human gastroenteritis, with the consumption of contaminated poultry meat as the main route of infection. Probiotic bacteria, such as *Lactobacillus*, *Bacillus*, *Escherichia coli* Nissle, and *Bifidobacterium* species, have a great immunomodulatory capacity and exhibit antipathogenic effects through various molecular mechanisms. Reducing *Campylobacter* levels in livestock animals, such as poultry, will have a substantial benefit to humans as it will reduce disease transmissibility through the food chain. Moreover, probiotic-based strategies might attenuate intestinal inflammatory processes, which consequently reduce the severity of *Campylobacter* disease progression. At a molecular level, probiotics can also negatively impact on the functionality of various *Campylobacter* virulence and survival factors (e.g., adhesion, invasion), and on the associated colonization proteins involved in epithelial translocation. The current review describes recent *in vitro*, *in vivo*, and preclinical findings on probiotic therapies, aiming to reduce *Campylobacter* counts in poultry and reduce the pathogen's virulence in the avian and human host. Moreover, we focused in particular on probiotics with known anti-*Campylobacter* activity seeking to understand the biological mechanisms involved in their mode of action.

## Introduction

Members of the genus *Campylobacter* are the utmost documented foodborne pathogens of the present century (Śmiałek et al, 2021). Being first isolated in 1963, *Campylobacter* is renowned for its exquisite capacity of adherence and biofilm formation on different surfaces, high invasion of various hosts, and for its increased viability outside of its natural biological niche (Erega et al, 2021a). Adhesion is one of the most important virulence factors required for *Campylobacter* survival during host–pathogen interaction being correlated with the animal and human infection rates (Šikić Pogačar et al, 2020). *Campylobacter jejuni* and *Campylobacter coli* are the most representative species of the *Campylobacter* genus known for their ability to form mono- or multispecies biofilms (Elgamoudi and Korolik, 2021).

These two species are responsible for causing 90% of the estimated human campylobacteriosis cases and are accountable for ≈84% (*C. jejuni*) and ≈9% (*C. coli*) of the total diagnosed cases in Europe (Soro et al, 2020). To successfully invade the human intestine and cause disease, the ingestion of only a few hundred bacterial cells is sufficient to initiate the *Campylobacter* infection, with an incubation period that varies from 24 to 72 h before the onset of illness (Soro et al, 2020). *Campylobacter-*induced disease is usually followed by postacute infection sequelae such as bacteremia, urinary tract infections, sepsis, and complicated immune-mediated neuropathies (Hayat et al, 2022) (e.g., Guillain–Barré and Miller-Fisher syndromes). These neuropathies are characterized by neuromuscular paralysis (Itamura et al, 2022), posing significant human health risks (Balta et al, 2021b; Elgamoudi and Korolik, 2021).

Poultry flocks are carriers of high levels of campylobacters (≈10^9^), which makes consumption of contaminated poultry meat the main source of infection in humans (Rawson et al, 2022). Henceforth, controlling *Campylobacter* levels at primary production represents a major step in preventing human infections; however, no single control method is yet capable of complete pathogen elimination (Hakeem and Lu, 2021). Probiotics are considered a viable tool for pathogen reduction (Cean et al, 2015) as they can limit the use of antibiotics and improve animal performance with the added benefit of antibiotic-free food products availability for human consumption (Sibanda et al, 2018). One of the probiotic positive impacts on gut microbiota is the endogenous production of short-chain fatty acids inducing immunostimulatory effects in farm animals (Melara et al, 2022).

**FIG. 1.**
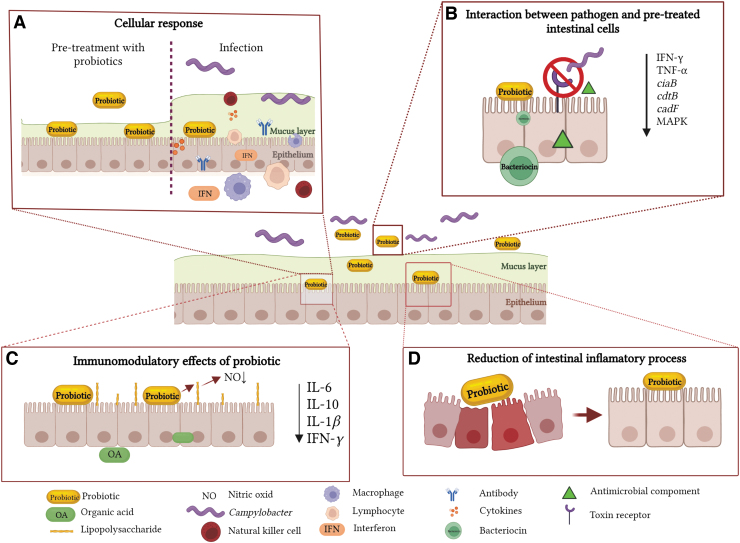
Probiotic mechanisms of action in alleviating *Campylobacter* infection. **(A)** Stimulation of cellular responses by inducing immunomodulatory effects and fortification of the GIT immunity through the production of interferon, and activation of specific antibodies, lymphocytes, macrophages, and NK cells that will relieve inflammation and mitigate *Campylobacter* infection. In addition, autoaggregation and coaggregation contribute to the competitive inhibition of *Campylobacter*. **(B)** Second, probiotics are associated with nutrient depletion in a specific environmental niche, obstructing the surface epithelial target receptors utilized by *Campylobacter* during infection. Probiotics will provide the molecular weaponry to outcompete and eliminate pathogens, colonize the environmental niche, secrete various antimicrobial components, and deploy enzymatic mechanisms responsible for modifying pathogen toxin receptors, hydrolysis of bacterial toxins, and inhibiting *Campylobacter*-induced infection of *Campylobacter* virulence factors. **(C)** Other beneficial immunomodulatory probiotic effects are the production of organic acids as antimicrobial molecules. Pretreatment of cells before the infection reduces the cytokine expression (IL-6, IL-10, IL-1β, and IFN-γ), and production of NO in LPS-stimulated cells. **(D)** Alleviates the intestinal inflammatory processes, which consequently will reduce the severity of the disease progression for the host. IFN, interferon; IL, interleukin; NK, natural killer; GIT, gastrointestinal tract; LPS, lipopolysaccharide; NO, nitric oxide. Figure created with *Biorender.com*.

Nevertheless, a more focused approach and a more profound understanding on how to standardize probiotic use in livestock and on their antipathogen efficacy are required. Based on the main effects of probiotics presented in Figure 1, in this study, we aim to discuss the latest advances in probiotic use as a strategy to reduce the pathogen virulence and transmissibility, in with a special focus on poultry but tangential to human infections.

## Probiotics and *Campylobacter* Colonization in Poultry

*Campylobacter* colonization of the avian gastrointestinal tract (GIT) initiates in the small intestines, and continues to the cecum and cloaca without any clinical manifestations of the disease (Dec et al, 2018; Śmiałek et al, 2021). Nevertheless, several reports have indicated that challenged chickens could experience focal hepatic necrosis, signs of disseminated hemorrhagic gastroenteritis, and jejunal distention (Workman et al, 2005). The natural *Campylobacter* infection occurs through the fecal–oral route and is being established in the cecum at levels of ∼10^9^ CFU/g of cecal content, with persistence during the entire bird lifetime (Awad et al, 2018; Dec et al, 2018). Once colonization is established, the entire flock becomes infected within a couple of days, with the infected birds showing excessive mucus production and enriched viscosity at an intestinal level (Awad et al, 2018).

It was also reported that higher levels of *Campylobacter* could be spotted in the crop region and with a lesser proportion in the broiler's gizzard (Smith and Berrang, 2006). In some cases, the *Campylobacter* spp. could also be detected in the internal organs (e.g., spleen, liver, bursa of Fabricius, and thymus), muscles, and blood samples during avian infection (Deng et al, 2020; Śmiałek et al, 2021). In response to infection, the avian host immunity triggers the production of proinflammatory cytokines, which modulate the GIT barrier function (Awad et al, 2018). The intestinal damage has a consequential impact on gut integrity by facilitating the transcellular/paracellular internalization of *Campylobacter* and progresses toward the underlying connective tissue, meanwhile eliciting the translocation of luminal pathogenic bacteria such as *Salmonella*, *E. coli*, and *Clostridium* to the internal organ compartments (Awad et al, 2018).

Nonetheless, can probiotics help in alleviating some of these effects? The biological mechanisms by which probiotics exhibit positive effects in poultry are not yet deciphered, but substantial advancements have been recently achieved in the case of *Campylobacter* spp. (Erega et al, 2021a; Kobierecka et al, 2017; Saint-Cyr et al, 2017). Details on the probiotic effects against *Campylobacter* spp. are in Table 1.

**Table 1.** Anti-*Campylobacter* Probiotics in Poultry

Probiotic	Concentration	Evidence	Gene/protein	Refs.
*In vivo*				
*Butyricicoccus pullicaecorum* 25–3^T^	10^9^ CFU lyophilized/kg	*Campylobacter* spp. reduction in cecum.	n.i.	Eeckhaut et al (2016)
*Lactobacillus salivarius* SMXD51	Suspension 10^7^ CFU	2.81 log reduction of *Campylobacter* in cecum.	↑ IL-8, β-defensin 2, and CXC chemokine (K60)	Saint-Cyr et al (2017)
Lavipan (*L. lactis*, *C. divergens*, *Lactobacillus casei*, *Lactiplantibacillus plantarum*, and *Saccharomyces cerevisiae*)		Lower *Campylobacter* spp. levels in GIT of birds, decreased environmental contamination, and increased meat hygiene.	n.i.	Smialek et al, (2018)
*S. cerevisiae boulardii* (CNCM I-1079)	1 × 10^9^ CFU/kg	Villi length and crypt depth, increased BW, lower *Campylobacter* spp. from fecal and cecal samples.	n.i.	Massacci et al (2019)
*Lactobacillus gallinarum* PL53	∼10^8^ CFU	Competitive exclusion, lower *Campylobacter jejuni* loads in cloacal and cecal swabs.	n.i.	Khan (2019)
Probiotic + OA	0.5 kg/ton	*In vivo*, 1.2 log reduction in *Campylobacter coli* in ceca.	n.i.	Mortada et al (2020)
*In vitro*				
*Lactobacillus* spp. (PCS20, PCS22, PCS25, LGG, PCK9)	1 × 10^8^ CFU/mL	Reduced *C. jejuni* adhesion, invasion, and translocation to chicken (B1OXI) and functional pig (PSI cl.1 and CLAB) cell line.	n.i.	Šikić Pogačar et al (2020)
*E. faecium*, *B. animalis*, and *P. acidilactici*	Dilution ratio of 1:1	Decreased gentamicin-resistant *C. coli* (CCGR) proliferation.	n.i.	Mortada et al (2020)
*L. reuteri*	Dilution ratio of 5:1	Inhibition of gentamicin-resistant *C. coli* (CCGR).		

BW, body weight; GIT, gastrointestinal tract; n.i., not identified.

The beneficial effects of probiotics are attributed to their ability to improve feed digestibility, nutrient bioavailability, and to enhance the immune system (Emami et al, 2020) and health leading to improved animal performance and carcass quality (Saint-Cyr et al, 2017; Yan and Polk, 2020). Most of the pathogen-associated inhibitory mechanisms of probiotics refer to nutrient depletion in a specific environmental niche, to the obstruction of the surface epithelial target receptors, usually used by pathogens during infection, and to their ability to synthesize natural antimicrobial molecules (Yan and Polk, 2020). The ability to induce potent immunomodulatory outcomes fortifies the avian-specific GIT immune mechanisms by producing interferon (IFN), antibodies, activated lymphocytes, macrophages, and natural killer cells to combat the diversity of infections and inflammatory processes upon *Campylobacter* infection (Śmiałek et al, 2021; Taha-Abdelaziz et al, 2019).

Clearly, probiotics have an immunomodulatory effect in poultry regardless of the pre- or posthatch administration as it has been shown that lactobacilli can modulate the immune response in newly hatched chickens (Alizadeh et al, 2021).

Organic acids were associated with antibacterial and immunomodulatory effects in poultry (Khan et al, 2022), also having an anti-*Campylobacter* effect (Sima et al, 2018). Butyrate-producing probiotic strains (e.g., *Butyricicoccus pullicaecorum*), improved feed efficiency and lowered *Campylobacter* levels in the ceca, by ∼1.5 logs, when fed to Ross 308 chicken broilers (Eeckhaut et al, 2016). Probiotic-derived butyrate also reduces the expression of proinflammatory cytokines (interleukin [IL]-6, IL-10, IL-1β, and IFN-γ) and hinders the production of nitric oxide in lipopolysaccharide (LPS)-stimulated cells (Trukhachev et al, 2021). Likewise, birds subjected to an oral gavage with a probiotic suspension of *Lactobacillus salivarius* SMXD51, 10^7^ CFU at 14 and 35 d, showed significant reductions (0.82–2.81 logs) in *Campylobacter* levels (Saint-Cyr et al, 2017). Furthermore, *L. salivarius* SMXD51, an effective producer of salivaricin, was previously associated with induced immunomodulatory effects by boosting the IL-8 production and increased secretion of β-defensin 2 (Saint-Cyr et al, 2017).

Probiotic beneficial effects are not only limited to lowering *Campylobacter* levels in the GIT, they also extend to reducing environmental contamination (e.g., farms), and ultimately contributing to enhanced carcass hygienic indices (Smialek et al, 2018). The positive impact in the GIT was illustrated when *C. jejuni-*challenged Ross 308 chicks were fed with 1 × 10^9^ CFU/kg *Saccharomyces cerevisiae boulardii* (CNCM I-1079). The significant improvement in villi height and crypt depth was accompanied by increased body weight gain and lower *Campylobacter* levels in the fecal and cecal samples (Massacci et al, 2019). Other similar broiler experiments concluded that *Lactobacillus gallinarum* PL53 (∼10^8^ CFU) has the ability to lower *C. jejuni* presence in the cloaca and ceca, emphasizing the potential application as inhibitors of *Campylobacter* colonization at primary production (Khan, 2019).

**FIG. 2.**
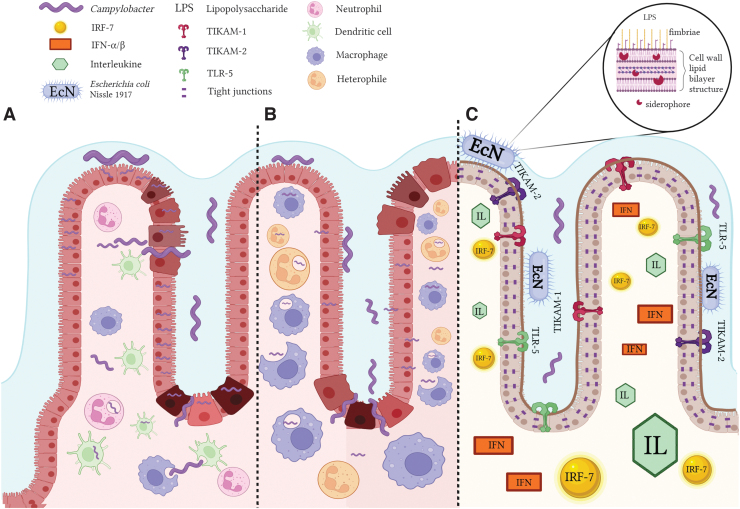
*Campylobacter* spp. infection of human and avian epithelium, and the proposed EcN mode of action against *Campylobacter* infection. **(A)** Infection provoked by *Campylobacter* in a human cellular model. **(B)** Infection of the chicken cell model. **(C)** Pretreatment with EcN prevents the infection induced by *Campylobacter* in an HT-29 cell infection model. EcN possesses fitness patterns and survival elements such as nonpathogenic adhesive fimbriae, siderophores, and a unique LPS that allows enhanced adherence to the epithelium. An immunomodulatory mechanism on HT-29 cells with the impact on *Campylobacter* infection occurs through the activation of IRF-7, TLR-5, and TLR adaptor molecules (TICAM-1 and TICAM-2), ILs, and IFN-α/β. The EcN pretreatment improved cellular barrier function by strengthening tight junctions, regulating the production of dendritic cells, enabling immune reactions through the synthesis of protecting immunoregulatory and proinflammatory cytokines and chemokines, extending the epithelial-cellular regeneration, and mitigating *Campylobacter-*induced cell apoptosis. IFN, interferon; TLR, toll-like receptor; IRF-7, interferon regulatory factors; LPS, lipopolysaccharide; EcN, *Escherichia coli* Nissle 1917. Figure created with *Biorender.com*.

Low numbers of campylobacters are able to also invade chicken intestinal cells (Fig. 2B) (Byrne et al, 2007), and thus, gaining knowledge on how probiotics can reduce colonization is beneficial and challenging. In summary, recent literature suggests that dietary supplementation of probiotics in poultry promotes an enforced intestinal barrier (Šikić Pogačar et al, 2020), enriches gut microbiota (Ty et al, 2022), lowers the systemic triglyceride and cholesterol levels (Vourakis et al, 2021), balances major blood biomarkers (e.g., albumin and glucose), and supports an enhanced absorption and digestion of the nutrients (Aponte et al, 2020). These benefits will contribute to disease prevention as well as to a reduced colonization of *C. jejuni* throughout infection (Dai et al, 2020).

## Probiotics and Preclinical Outcomes in Human *Campylobacter* Infections

In humans, oral and intestinal colonization (Lee et al, 2022) represents the first stages of *C. jejuni* pathogenesis (Fig. 2A) and is usually followed by distending toxin-mediated enteric infection and campylobacteriosis (Upadhyay et al, 2019). Several other findings have also established that the *Campylobacter* spp. isolated from the human oral cavity are indeed correlated with intestinal disease, and, in some instances, the detection levels are higher than in the fecal or intestinal biopsies (Xu et al, 2021). In addition, oral health studies have reported that *Campylobacter* abundance was slightly increased in the oral cavity of individuals encountering periodontal diseases and dental caries comparing with healthy patients (Lif Holgerson et al, 2015; Xu et al, 2021).

With the consumption of contaminated poultry meat being the main source of infection in humans, reducing *Campylobacter* levels in poultry through dietary probiotics could equally reduce human exposure to *Campylobacter* (Šikić Pogačar et al, 2020; Taha-Abdelaziz et al, 2019). Although previously thought that probiotic-based therapies are still marginalized in the absence of extensive clinical research, however, several recent findings have reported that probiotics are implicated in attenuating *Campylobacter* infection severity and are listed in Table 2. Probiotics can create a molecular weaponry necessary to outcompete and eliminate pathogens, colonize the environmental niche, and secrete various antimicrobial components. They can deploy enzymatic mechanisms responsible for modifying pathogen toxin receptors, hydrolysis of bacterial toxins, and inhibition of pathogen-induced illness (Trukhachev et al, 2021).

**Table 2.** Anti-*Campylobacter* Probiotics, Evidence at Preclinical Level

Probiotic	Concentration	Evidence	Gene/protein	Refs.
*In vitro*				
*Bifidobacterium longum* subsp. *infantis* ATCC 15697 and goat milk oligosaccharides	5 mg/mL	Reduced *Campylobacter jejuni* 81–176 invasion and adhesion of HT-29 cells.	n.i.	Quinn et al (2020)
*Lactobacillus gasseri* SBT2055	1 × 10^8^ CFU 200/μL	Inhibits adhesion/coaggregative phenotype, cell surface aggregation—promoting factors (APF1) responsible for the competitive exclusion.	↑ APF	Nishiyama et al (2015)
*Lactobacillus* spp. mixture (*L. crispatus*, *L. johnsonii*, *L. salivarius*, and *L. gasseri*, *L. reuteri*)	10^8^ CFU	Immunomodulatory activity, decreased virulence-associated factors, blocked production of *C. jejuni* quorum-sensing autoinducer-2, and reduced *in vitro* invasion in Caco-2 cells.	↓ *ciaB*, *flhA*, *flaA*, *flab*, *luxS*, ↑ IL-1β, IL-10, IL-12p40, CXCLi2, CD40, CD80, and CD86	Taha-Abdelaziz et al (2019)
Cell-free culture supernatants of genetically modified *L. casei*	2 × 10^6^ CFU/mL	Reduced bacterial growth, invasion and adherence of HeLa and HD-11 cells.	↓*ciaB*, *cdtB*, *cadF*, ↑ flaB	Tabashsum et al (2018)
Preclinical				
Aviguard^®^ formulation	1 g dissolved in 10 mL of PBS and each subject perorally received 0.3 mL of the bacterial suspension, 10^9^ CFU/g	*In vivo* disease-alleviating effects by attenuating the apoptotic cell responses from the *C. jejuni* 81–176-infected large intestine sections.	↓ IFN-γ, TNF-α	Heimesaat et al (2021)
*L. johnsonii*	10^8^ CFU	Immunomodulatory effects in *C. jejuni* 81–176-infected C57BL/6j mice.	↓ NO, IL-6, IL-10, TNF, NOD	Bereswill et al (2017)
VSL3 mixture	10^9^ Viable bacteria	Reduced intestinal apoptosis and proinflammatory immune responses.	↓TNF, IL-12p70, IL-6, MCP-1, ↑IL-10	Ekmekciu et al (2017)
*L. plantarum* LP5	8.78 log/CFU	Reduced *C. coli* in feces, cecum, and ileum of DSPV458 infection mouse model.	n.i.	Ruiz et al (2021)

APF, antiproliferative factor; n.i., not identified; NO, nitric oxide; PBS, phosphate-buffered saline; IL, interleukin; MCP-1, monocyte chemoattractant protein-1; TNF, tumor necrosis factor.

The competitive exclusion skill of probiotics could be enhanced with synbiotics, as it was recently exemplified that *Bifidobacterium longum* subsp. *infantis* ATCC 15697 and goat milk oligosaccharides prevented *C. jejuni* 81–176 invasion and adhesion of HT-29 cells (Quinn et al, 2020).The competitive exclusion includes probiotic migration to the adhesion sites, more fastidiously and rapidly, hence arriving at the adhesion sites quicker than *C. jejuni* (Šikić Pogačar et al, 2020). For example, the competitive inhibition of *Campylobacter* by lactobacilli includes mechanisms such as autoaggregation, coaggregation with the pathogen itself, and competition for the attachment sites (Nishiyama et al, 2014). Exclusion led to a significant reduction in the number of *C. jejuni*, which adhered and invaded human and avian epithelial cells, while the reductant coaggregation was associated with the production of specific proteinaceous surface compounds, which together could be involved in the mitigation of *C. jejuni* colonization and infection.

Recent preclinical findings reported that during *C. jejuni*-induced murine enterocolitis, the per-oral administration of a commercial probiotic (Aviguard) attenuated the apoptotic cell responses in *C. jejuni* 81–176-infected large intestine and improved the clinical outcomes (Heimesaat et al, 2021). The investigation of colonic biopsies, at 6 d postinfection, indicated statistically lower levels of IFN-γ and tumor necrosis factor alpha (TFN-α) after Aviguard treatment compared with the placebo groups. Meanwhile, the probiotic treatment averted *C. jejuni*-induced IFN-γ secretion from extraintestinal organs such as the liver, lungs, and kidneys. Lastly, an interesting fact from this pre-clinical trial indicated that the probiotic suspension Aviguard was successfully recovered from the intestines of treated subjects (Heimesaat et al, 2021) indicating its survival within the gut microbiome.

Recent data suggest that *Caenorhabditis elegans* activates the antibacterial peptide genes, including the upregulation of Daf-16 transcription factor and of MAPK signaling pathways, as an immunogenic effect against *C. jejuni* (Jin et al, 2021). Similarly, genetically engineered lactic acid-producing strains, *Lactobacillus casei* (LC+mcra), have the capacity to generate higher concentrations of conjugated linoleic acid, which showed a statistically high efficiency in *C. jejuni* growth decline (Tabashsum et al, 2018). Under coculturing conditions, the modified *L. casei* and its cell-free culture supernatants reduced the invasion and adherence of *C. jejuni* to HeLa and HD-11 cell lines. Some of *C. jejuni* virulence, including *cia*B, *cdt*B, and *cad*F genes, was significantly downregulated in contrast to the *fla*B gene, which was significantly upregulated (Tabashsum et al, 2018).

In mice, using unconventional preclinical interventions, based on human fecal microbiota transplantation (FMT), per-oral treatment with probiotic strains provided evidence of the effective decrease in *C. jejuni* 81–176 levels and led to the mitigation of systemic inflammation and reduced pathogen-induced intestinal sequelae (Heimesaat et al, 2019a; Heimesaat et al, 2019b). Conclusively, it was suggested that novel probiotic formulations, as alternative strategies to FMT during severe GIT inflammations, might be efficient in lowering pathogen levels in vertebrates and farm animals and could even treat campylobacteriosis (Heimesaat et al, 2019b). Taken together, these studies indicate that clinical trials are the next obvious step in elucidating the *in vivo* effects of probiotics in preventing campylobacteriosis or in alleviating its secondary effects.

## Types of Probiotics Used Against *Campylobacter* spp.

In the absence of virulence factors and with a boost in fitness patterns and survival elements, *E. coli* Nissle 1917 (EcN) (Fig. 1) represents the best example of a potent probiotic with a unique LPS that attributes immunogenicity without triggering immunotoxicity (Mawad et al, 2018). The biological safety of EcN was previously investigated, in both animal and human trials, with promising results in mitigating human ulcerative colitis, diarrhea, and other inflammatory-related diseases (Balta et al, 2021a; Garrido-Mesa et al, 2011; Mawad et al, 2018; Scaldaferri et al, 2016). Moreover, EcN was involved in the upregulation of intestinal antioxidant and anti-inflammatory reactionsm, which improved the antipathogen effect in poultry and mammalian cells and reduced diarrheal infection (Garrido-Mesa et al, 2011; Mawad et al, 2018). Alongside other recent *in vitro* findings, the EcN inhibitory activity against *Campylobacter* is further detailed in Figure 2 and Table 3.

**Table 3.** *In Vitro* Evidence of Probiotics Efficient Against *Campylobacter*

Probiotic	Concentration	Evidence	Gene/protein	Refs.
*Escherichia coli Nissle* 1917	1 × 10^7^ CFU	Decreased *Campylobacter jejuni* 81–176 invasion and survival in HT-29 cells. Improved barrier functions and tight junction integrity of intestinal epithelial cells.	↑Claudins, cadherins/catenins, actinins	Helmy et al (2017)
*E. coli* Nissle 1917 microencapsulation in alginate-chitosan nanoparticles	2 × 10^8^ CFU	Reduced *C. jejuni* 81–176 [ATCC^®^ BAA-2151™] intracellular survival and invasion in HT-29 cells.	↓IL-12A, IL-1B, IL-18, CXCL8, TNF, TLR-1, TLR-4, and TLR-6	Mawad et al (2018)
*E. coli Nissle* 1917	1 × 10^7^ CFU	Modulates the immune responses, antiapoptotic Akt signaling protecting against the proinflammation of *C. jejuni* 81–176.	↓ IL-6/8/18, IL12-B, TNF, NF-κB, MAPK-1/3/8/14, MAP2K3, TLR, TICAM-1, TICAM-2, NOD-1, CASP-8, RIPK-2, JUN, IRAK-3, TRAF-6	Helmy et al (2021)
*L. curvatus DN317* with 28 mM glycerol	50, 100, and 150 AU/mL	Curvaticin DN317 production is bacteriostatic against *C. jejuni* ATCC 33560	n.i.	Zommiti et al (2016)
*L. reuteri* PTA5_F13	1.4 mM	Reuterin reduced *C. jejuni* from 7.3 log CFU/mL to the above detectable limits.	n.i.	Asare et al (2020)
*B. subtilis* PS-216	1:1, 1:10	*Bacillaene* reduces *C. jejuni* NCTC11168 growth by ≈4.2 logs, biofilm formation, and adhesion based on diffusible factors.	n.i.	Erega et al (2021a); Erega et al (2021b)

n.i., not identified; NF-κB, nuclear factor kappa B; TLR, toll-like receptor.

As described in Figure 2C, the immunomodulatory effects of EcN, with impact on *C. jejuni* infection, include enabling of the nuclear factor kappa B (NF-κB) signaling pathways, the interferon regulatory factors (IRF-7), and the toll-like receptor (TLR) adaptor molecules (TICAM-1 and TICAM-2). In the HT-29 model of infection, the EcN modulatory effects extend to TLR-4 signaling, IL-12A/B, IL-1B, IL-17A, IFN-α/β, and the extracellular signal-regulated kinase pathway (ERK-1 and ERK-2), p38MAPK, antiapoptotic Akt signaling, and the c-Jun-NH2-kinase (JNK) (Helmy et al, 2021). Another interesting observation was that the expression of proinflammatory cytokines (IL-6/8/18, IL12-B, and TNF), NF-κB, mitogen-activated protein kinases (MAPK-1/3/8/14 and MAP2K3), TLR and TLR adaptor molecules (TLR-4/TLR-5, TICAM-1, and TICAM-2), NOD-1, apoptosis regulating factors (CASP-8 and RIPK-2), JUN, and MYD88-related (IRAK-3 and TRAF-6) genes was downregulated when cells were pretreated with EcN before *C. jejuni* infection (Helmy et al, 2021).

These mechanistic insights are linked to prestimulation of the epithelial immune protection systems to facilitate counteraction of the proinflammatory response caused by *C. jejuni* infection. Overall, these results improved our knowledge on how probiotic agents, such as EcN, interrupt *C. jejuni* infection and gastroenteritis, prevent disruption of the epithelial barrier, and inhibit the host's proinflammatory responses (Helmy et al, 2017; Helmy et al, 2021; Mawad et al, 2018).

Other means by which probiotics exclude bacterial pathogens include the production of bacteriocins, efficient antimicrobial peptides (Ahsan et al, 2022). One example is curvaticin, a bacteriocin synthesized by *Lactobacillus curvatus* DN317, which expressed a remarkable bacteriostatic activity against the chicken isolate *C. jejuni* ATCC 33560 (Zommiti et al, 2016). Growth inhibition was achieved when 50, 100, and 150 AU/mL of *L. curvatus* supernatant was introduced to the growing culture of *C. jejuni* ATCC 33560 and decreased the viable bacterial counts by ≈23.8%, 45.5%, and 61.3%, respectively (Zommiti et al, 2016).

Enterocins are another example of bacteriocins, derived from *Enterococci* spp., which in combination with herbal extracts can be applied as a new ecological approach to *Campylobacter* reduction in livestock commodities, therefore improving human health safety (Ščerbová et al, 2022). *Lactobacillus reuteri* could also produce inhibitory compounds, under anaerobic fermentation conditions, such as reuterin, which has broad-spectrum antimicrobial activity against various foodborne pathogens (Asare et al, 2020). A chicken-synthesized reuterin, produced by *L. reuteri* PTA5_F13, demonstrated a more significant inhibition of *C. jejuni* N16-1419 when 28 mM glycerol, added in the *in vitro* experimental conditions, was used as a reuterin precursor (Asare et al, 2020).

Furthermore, the study results showed that during coculturing of *L. reuteri* PTA5_F13 with 28 mM glycerol, *C. jejuni* counts drastically dropped from 7.3 logs CFU/mL to just above the detectable limits (1 log CFU/mL). The study concluded that such efficient anti-*Campylobacter* outcomes might be valuable in slaughterhouse poultry equipment decontamination processes.

Biosurfactants, another category of antiadhesive molecules (e.g., fengycin, iturin, and surfactin, produced by *Bacillus subtilis*) (Erega et al, 2021a), have the ability to significantly reduce *C. jejuni* NCTC11168 (by ≈4.2 log_10_) growth, and biofilm formation and adhesion to abiotic polystyrene surfaces, through a mechanism dependent on diffusible factors (e.g., nonribosomal/polyketide bacillaene) (Erega et al, 2021a). Promising anti-*Campylobacter* effects of *B. subtilis* PS-216 were also outlined in several other studies (Erega et al, 2021a; Šimunović et al, 2022). First, *B. subtilis* PS-216 demonstrated significant antagonism against *C. jejuni* through growth inhibitory effects (4.2 log_10_) and reduced biofilm formation and adherence (2.4 log_10_) to abiotic surfaces. These effects were attributed to the production of the antimicrobial compound nonribosomal/polyketide bacillaene (Erega et al, 2021a). The authors specified that wild-type *B. subtilis*, carrying the *sfp* gene encoding for phosphopantetheinyl transferase, is linked to the production of antimicrobial molecules and to the expression machinery of bacillaene.

Second, it was reported that under coculture conditions, *B. subtilis* reduced *C. jejuni* growth by 3.87–4.07 logs, and dropped below the detection limits after 48 h (Šimunović et al, 2022). Given that all these effects were observed at 42°C, which is the optimal body temperature in poultry, *B. subtilis* PS-216 becomes a strong probiotic candidate for *Campylobacter* reduction in poultry (Šimunović et al, 2022).

Efficient probiotics will also have to be able to overcome and survive the GIT environment (Grispoldi et al, 2020). Probiotic candidates in poultry, such as *Lactobacillus* spp., have shown adhesion and invasion inhibitory properties, against *C. jejuni* K49/4, and persistence in the chicken small-intestine cells and pig enterocytes (Šikić Pogačar et al, 2020). Coculturing of *C. jejuni* with *Lactiplantibacillus plantarum* PCS20, PCS22, PCS25, and PCK9 considerably declined the number of campylobacters that adhered and invaded the polarized intestinal epithelial cells. Compared with other strains, *Lactobacillus rhamnosus* LGG and *L. plantarum* PCS25 statistically reduced the invasion of *C. jejuni* below the limit of detection (Šikić Pogačar et al, 2020). *L. plantarum* was also efficient in broilers when part of a microencapsulated symbiotic was combined with fructooligosaccharides, having a positive impact on growth performance, immune and antioxidant parameters, and the digestibility of calcium and phosphorus (Song et al, 2022).

*In vivo* has been confirmed that lactic acid bacteria can reduce *C. jejuni* NCTC 11168 pathogenicity, first in a *C. elegans* nematode model followed by subsequent extrapolation into a mouse and chicken model (Jin et al, 2021). When nematodes were infected with *C. jejuni* and administered *Lactobacillus* spp. (13-7, N9, and Z5 strains), a significant increase in the expression of MAPK signaling pathway genes (*pmk*-1, *nsy*-1, *sek*-1, and *tir*-1), antioxidant genes (*skn*-1 with *bar*-1), defence immune genes (*daf*-16, *age*-1, and *dbl*-1), and antibacterial peptide genes (*spp*-1, *clec*-85, *abf*-2, *clec*-60, and *lys*-7) was observed (Jin et al, 2021). Strain Z5 also demonstrated increased inhibitory activity of *C. jejuni* colonization in the chicken ceca (below 10^4^ CFU/g), in contrast to the untreated control and the infected group where counts were above 10^8^ CFU/g.

Less efficient, but significant, decreasing trends were also reported for the 13-7 and N9 isolates, which lowered the ceca counts by ∼10^3^ CFU/g. The combined effects of all the above examples strengthen the general view that the *C. jejuni*-antagonistic effects of probiotics are articulated by the overexpression of the immune-associated genes, as proven and previously described in nematode, mice, and chicken models of infection.

## Conclusion

The findings of this review describe the latest antivirulence, broad antibacterial, and immunomodulatory effects of various bacterial probiotic species against emerging *Campylobacter* spp. infections. The anti-*Campylobacter* probiotics will potentially possess other desired features (e.g., acidic/bile resilience, bile salt hydrolase capacity, efficient colonization of the gut properties) indicating wider health beneficial effects and potent immunomodulatory effects. More efforts should be made to further elucidate and decipher the pathogenic mechanisms of enteropathogenic bacteria such as *Campylobacter*, especially in poultry, to accelerate the validation and accreditation of new probiotic strains.
